# Exaggerated IL-17A activity in human in vivo recall responses discriminates active tuberculosis from latent infection and cured disease

**DOI:** 10.1126/scitranslmed.abg7673

**Published:** 2021-05-05

**Authors:** Gabriele Pollara, Carolin T Turner, Joshua Rosenheim, Aneesh Chandran, Lucy CK Bell, Ayesha Khan, Amit Patel, Luis Felipe Peralta, Anna Folino, Ayse Akarca, Cristina Venturini, Tina Baker, Simone Ecker, Fabio LM Ricciardolo, Teresa Marafioti, Cesar Ugarte-Gil, David AJ Moore, Benjamin M Chain, Gillian S Tomlinson, Mahdad Noursadeghi

**Affiliations:** 1 University College London, London, UK; 2 School of Medicine, Universidad Peruana Cayetano Heredia, Lima, Peru; 3 Department of Clinical and Biological Sciences, University of Turin, Turin, Italy; 4 TB Centre, London School of Hygiene & Tropical Medicine, London, UK; 5 Laboratorio de Investigación de Enfermedades Infecciosas, Universidad Peruana Cayetano Heredia, Lima, Peru

## Abstract

Host immune responses at the site of *Mycobacterium tuberculosis* (Mtb) infection can mediate pathogenesis of tuberculosis (TB) and onward transmission of infection. We hypothesized that pathological immune responses would be enriched at the site of host-pathogen interactions modelled by a standardized tuberculin skin test (TST) challenge in patients with active TB compared to those without disease, and interrogated immune responses by genome-wide transcriptional profiling. We show exaggerated interleukin (IL)-17A and Th17 responses among 48 individuals with active TB compared to 191 with latent TB infection, associated with increased neutrophil recruitment and matrix metalloproteinase-1 expression, both involved in TB pathogenesis. Curative antimicrobial treatment reversed these observed changes. Increased IL-1β and IL-6 responses to mycobacterial stimulation were evident in both circulating monocytes and in molecular changes at the site of TST in individuals with active TB, supporting a model in which monocyte-derived IL-1β and IL-6 promote Th17 differentiation within tissues. Modulation of these cytokine pathways may provide a rational strategy for host-directed therapy in active TB.

## Introduction

*Mycobacterium tuberculosis* (Mtb) infection results in a spectrum of clinical outcomes, from asymptomatic latent infection to symptomatic disease. The focus of host-pathogen interactions is characterized histologically by granulomatous inflammation that may restrict bacterial growth, but this response can also result in tissue damage that promotes transmission of infection to other individuals (1, 2). The distinctions that tip the balance between protective and pathogenic immune responses remain a fundamental question in tuberculosis research. This knowledge is expected to inform rational vaccine design by identifying correlates of disease protection. However, understanding the molecular components that drive immunopathology can also guide development of host-directed therapies that attenuate tissue damage, limit morbidity and mortality, and reduce onward transmission. In addition, this approach may provide an adjuvant to antimicrobial therapy, allowing reduced duration of antimicrobial treatment and mitigating against increasing antimicrobial resistance (3).

Chronic inflammatory pathology at the site of human tuberculosis (TB) infection has been the subject of extensive descriptive studies, but discriminating between protective and pathological immune responses has been limited to comparing leukocyte phenotype and function in blood from Mtb-exposed patients with and without active disease (1). We have shown that genome-wide transcriptional profiling of biopsies of the tuberculin skin test (TST) can be used to make comprehensive molecular and systems level assessment of in vivo immune responses at the site of standardized host-pathogen interactions, including Mtb-infected lungs and lymph nodes (4–7). Importantly, the transcripts enriched within TST biopsies reflected the genome-wide variation in molecular pathology at the site of TB disease (5, 7), suggesting that the TST represents a valuable surrogate for assessing TB immunopathogenesis in humans in vivo.

In the present study we aimed to test the hypothesis that immune responses at the site of host-pathogen interactions, modelled by the TST, would reveal immunopathologic responses in patients with active TB that were absent in individuals with equivalent immune memory for Mtb but without disease. We used in vivo transcriptional responses at the site of TST to deconvolute differential cellular infiltration and functional cytokine activity, identifying in active TB the enrichment of molecular changes associated with immunopathology. We supplemented these findings with cytokine responses from ex vivo-stimulated monocytes to derive a mechanistic model of disease pathogenesis centered around elevated interleukin (IL)-17A activity as a driver of tissue damage in TB disease, in turn identifying a putative target for host-directed interventions.

## Results

### Immune responses at the site of TST in active and latent TB

**Figure 1:**
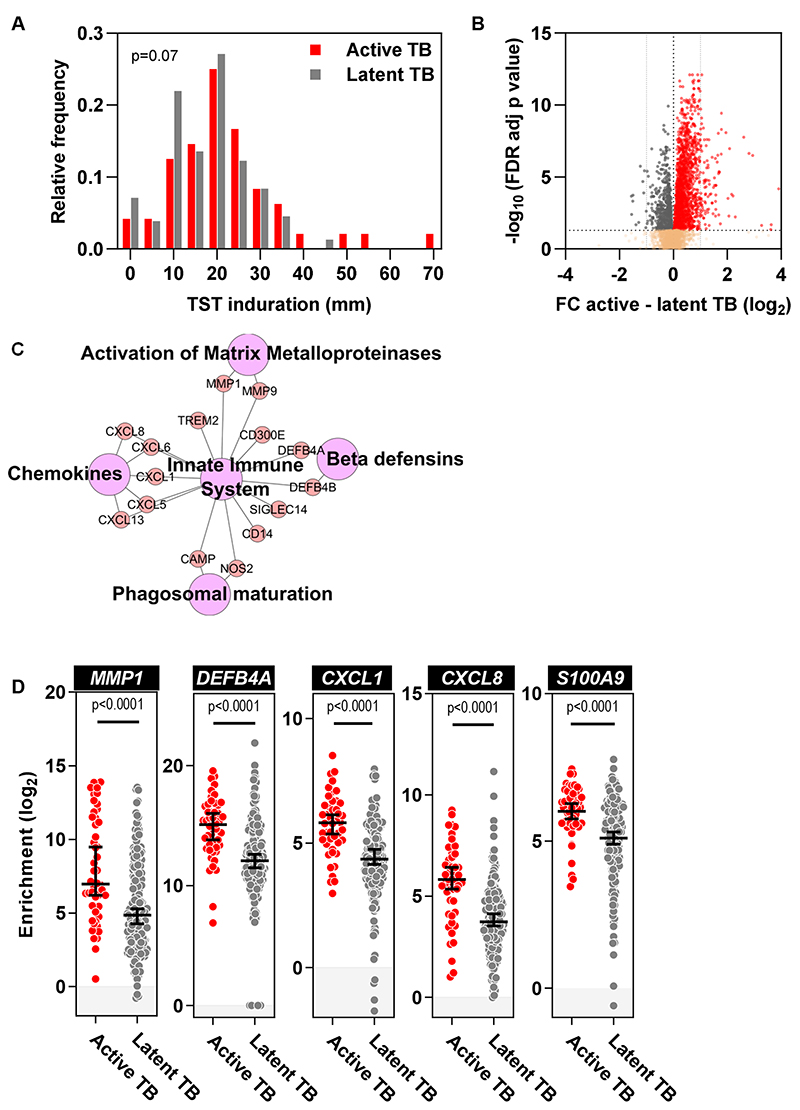
Enriched responses in the TST transcriptome of patients with active TB disease. **(A)** Induration at the site of TST was measured in millimeters and recorded by routine clinical assessments in both populations. **(B)** Volcano plot depicting relationship between relative fold change (FC) in gene expression between active and latent TB and statistical significance (-log_10_ FDR adjusted p value). Red dots represent genes with significantly greater expression in active compared to latent TB. Grey dots represent the converse, with greater expression in latent TB. **(C)** Network diagram of Reactome pathways and genes over-expressed with > 1 log_2_FC in the TST of patients with active TB compared to latent TB. Purple nodes represent Reactome database functional pathways, red nodes represent genes and edges reflect relationship between pathways and genes. Pathway node diameters are proportional to the respective pathway –log10 p value enrichment statistic. **(D)** Enrichment of selected gene transcripts within the TSTs of patients with active or latent TB relative to a separate cohort of individuals receiving a saline injection. Analyses were performed on samples from 48 and 191 participants with active or latent TB, respectively. Horizontal lines and error bars on scatter dot plots represent medians with 95% confidence interval. All p values were calculated by Mann-Whitney tests. *MMP1*, Matrix Metallopeptidase 1; *DEFB4A*, Defensin Beta 4A; *CXCL1*, C-X-C Motif Chemokine Ligand 1; *CXCL8*, C-X-C Motif Chemokine Ligand 8; *S100A9*, S100 Calcium Binding Protein A9.

The TST has been extensively used to identify patients with T cell memory for mycobacterial antigens, but the magnitude of the clinical response does not differentiate infected individuals with and without active TB disease (1). We sought to test the hypothesis that molecular profiling of the TST may identify elements of the recall response that are specifically associated with disease. We undertook 48-hour TSTs in 48 human immunodeficiency virus (HIV)-negative patients with microbiologically-confirmed pulmonary TB disease within the first month of treatment (‘active TB’, cohort 1) to identify disease-associated responses. We compared these to TST responses in 191 HIV-negative patients with latent TB, representing non-disease associated recall responses. The latent TB cohort was defined by a positive peripheral blood interferon gamma release assay (IGRA) and absence of clinical and radiological evidence of active TB disease (table S1). The active TB cohort was moderately over-represented by male recruits, whereas Asian patients were more frequent in the latent cohort. However, the age distribution was comparable between the two groups (Table 1). As expected, clinical induration in response to the TST was not different between the two groups (Fig. 1A), hitherto interpreted to reflect comparable cell-mediated immune memory.

In comparison to skin biopsies from the site of control saline injection, 7065 genes showed increased expression in response to the TST in at least one study group (Data File S1). Of these, 5743 were present in both groups (fig. S1A). Systems level assessment of the shared response revealed the prototypic cell mediated immune responses which we had previously described in the TST (fig. S1B) (5). We have previously shown that molecular changes in the TST reflect closely host responses at the site of TB disease (5, 7), and we extended these analyses by demonstrating the shared TST response to be enriched in the wall of Mtb-infected cavities relative to both healthy lungs and macroscopically normal adjacent lung tissue sections (8) (fig. S1C). Pairwise assessment of expression of the integrated list of transcripts that were enriched in either patient group showed these to be correlated, consistent with the hypothesis that at the level of individual molecules, most responses do not discriminate between the two groups (fig. S1D).

A proportion of transcripts were differentially enriched between the two groups (Fig. 1B, Data File S1). We focused on 151 of these genes that were expressed more than twofold higher in patients with active TB compared to patients with latent TB (Fig. 1B and C). Amongst these, Reactome Pathway Analysis identified enrichment for matrix metalloproteinases (MMPs), such as MMP-1, that has been strongly implicated in pathogenic degradation of the extracellular matrix in TB (9), as well as for beta defensins that exert both antimicrobial functions and also provide a chemotactic gradient for CCR2-expressing cells, including neutrophils (10) (Fig. 1D). The TST in active TB was also enriched for other genes that are chemotactic for neutrophils, such as *CXCL1*, *CXCL8* and *S100A9* (Fig. 1D) (11, 12).

### Elevated IL-17A responses in active TB

**Figure 2:**
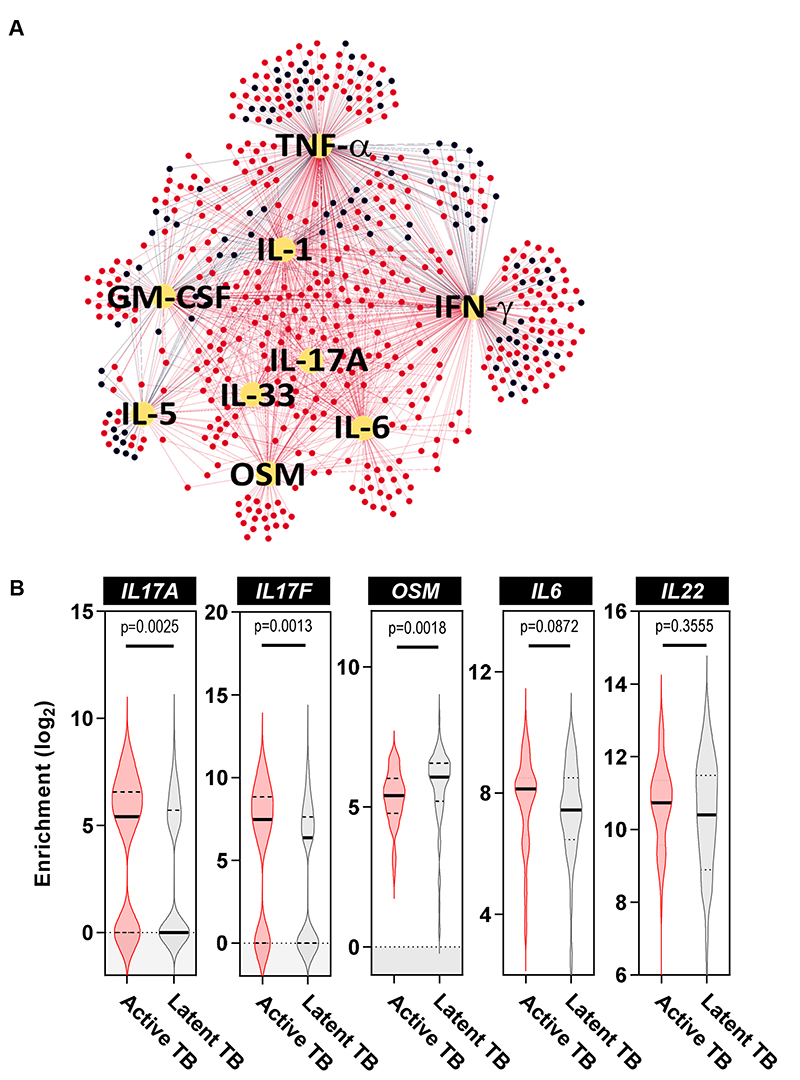
IL-17 cytokines are over-expressed at the site of TST in individuals with active TB. **(A)** Network diagram depicting upstream cytokine analysis of genes differentially expressed in TST of patients with active and latent TB. Red and black nodes represent significant genes overexpressed in active and latent TB, respectively. Yellow nodes represent predicted cytokines regulating the gene expression of red and black nodes. Edges depict relationship between upstream regulators and differentially expressed genes. IL, interleukin; IFN, interferon; TNF, tumor necrosis factor; GM-CSF, Granulocyte-Macrophage Colony-Stimulating Factor; OSM, Oncostatin M **(B)** Enrichment of selected cytokine transcript within the TSTs of active and latent TB patients relative to saline injection. Analyses performed on samples from 48 and 191 participants with active and latent TB, respectively. Violin plots represent frequency distribution of all samples, with bold and dashed lines representing median and quartile values. All p values were calculated by Mann-Whitney tests. *IL17A*, Interleukin 17A; *IL17F*, Interleukin 17F; *OSM*, Oncostatin M, *IL6*, Interleukin 6; *IL22*, Interleukin 22.

We hypothesized that the genes over-expressed in active TB were regulated by common upstream signals in the tissue environment. To test this hypothesis, we compared the predicted upstream regulators of enriched transcripts in the TST of active and latent TB patients using Ingenuity Pathway Analysis (6). This analysis suggested that interferon (IFN)-γ, tumor necrosis factor (TNF)-α and IL-1 family cytokines were predicted to be upstream signals for genes enriched in both active and latent TB. In contrast, IL-17A, oncostatin M (OSM) and IL-6 were predicted to induce the expression of genes uniquely enriched in active TB (Fig. 2A). We hypothesized that the predicted upstream signals would also be enriched at the gene level in the TST of patients with active TB. IL-17A and IL-17F, a closely related member of this cytokine family that binds the same receptor and exerts similar functions, were enriched in active TB (Fig. 2B) (11). In contrast, we found no difference in the expression of OSM or IL-6 transcripts (Fig. 2B). We also evaluated expression of IL-22, representing another cytokine with closely related biological function to IL-17A and IL-17F (13), but this was not differentially enriched in active TB (Fig. 2B).

**Figure 3:**
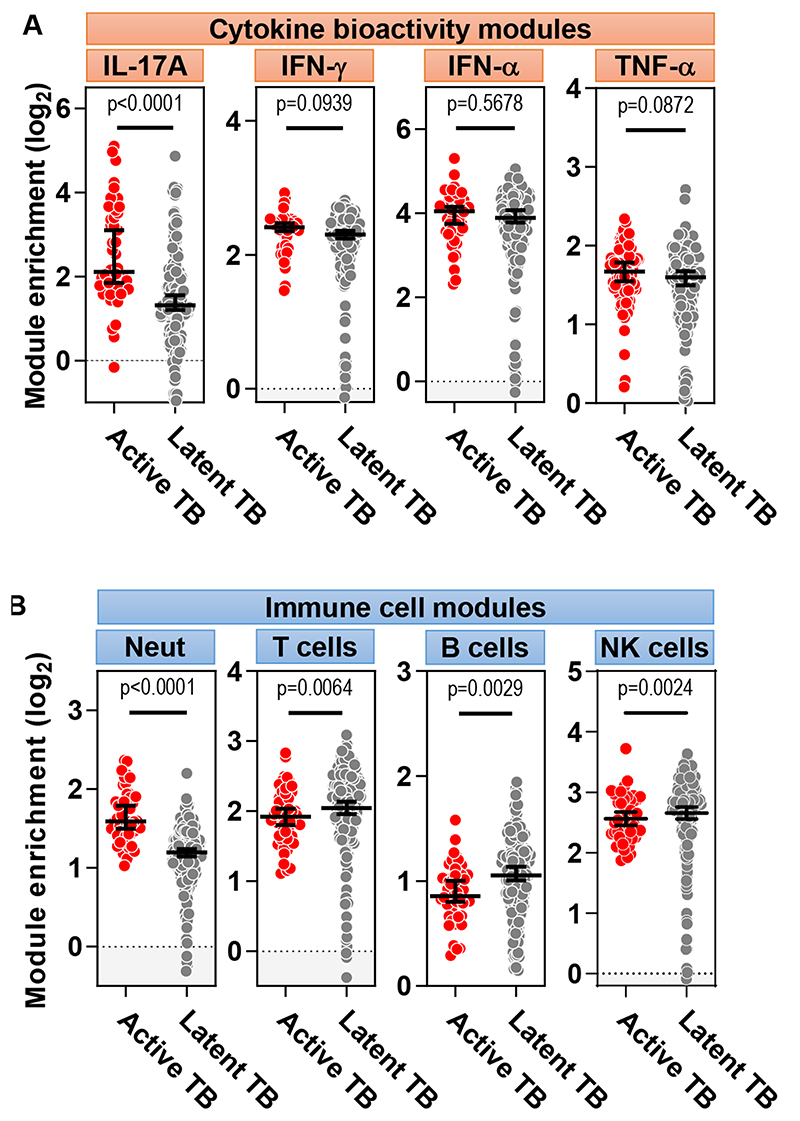
TST challenge in active TB is characterized by enrichment of IL-17A response and neutrophil-associated transcriptional modules. **(A)** Analysis of gene expression from TST samples revealed enrichment in keratinocyte response modules of cytokine bioactivity. IL, interleukin; IFN, interferon; TNF, tumor necrosis factor. **(B)** Modules for neutrophils (Neut), T cells, B cells and NK cells revealed enrichment of neutrophil-associated genes in TST samples from individuals with active TB. Analyses were performed on samples from 48 and 191 participants with active and latent TB, respectively. Horizontal lines and error bars on scatter dot plots represent medians with 95% confidence interval. All p values were calculated by Mann-Whitney tests.

To further test the functional relevance of the differences in IL-17A/F expression between active and latent TB, we extended our analyses to look more broadly at genes downstream of IL-17A. We derived gene expression modules for the specific transcriptional response of keratinocytes (KC) to IL-17A or other cytokines (14, 15) (Data File S2). We used the geometric mean expression of these modules as a biosignature to quantify cytokine activity *in vivo*. We confirmed that the resulting transcriptional modules were both sensitive and specific for their cognate stimuli by assessing their expression in other independent datasets (fig. S2). We then compared the expression of these cytokine-specific transcriptional modules in the TST transcriptomes as a measure of the functional activity of each cytokine. The IL-17A-induced gene module was increased in the TST of people with active TB compared to that of latent TB, but expression of IFN-γ, IFN-α, or TNF-α inducible gene modules was not different between the two groups (Fig. 3A).

A key function of IL-17A/F is to promote neutrophil recruitment via induction of neutrophil chemokines (16). Importantly, neutrophils also contribute to the immunopathology of TB (12, 17–20). Therefore, we tested the hypothesis that the TST in active TB would reveal increased neutrophil recruitment, compared to that of patients with latent TB. Consistent with this hypothesis, we found higher expression of a neutrophil transcriptional module, previously validated as a measure of the number of neutrophils (21), in the TST transcriptome of people with active TB compared to people with latent TB (Fig. 3B). We verified this enrichment of neutrophils in active TB using an independent immune cell deconvolution strategy (22) (fig. S3), a finding which was consistent with the higher gene expression of the IL-17A-inducible chemokines CXCL1, CXCL8 and S100A9 in active TB that promote neutrophil recruitment to sites of inflammation (11, 12) (Fig. 1D). In contrast, accumulation of T, B, and natural killer (NK) cells, quantified by expression of their respective transcriptional modules, (21), was greater in the TST of patients with latent TB compared to active TB (Fig. 3B).

### Increased frequency of Th17 cells in active TB TST responses

**Figure 4:**
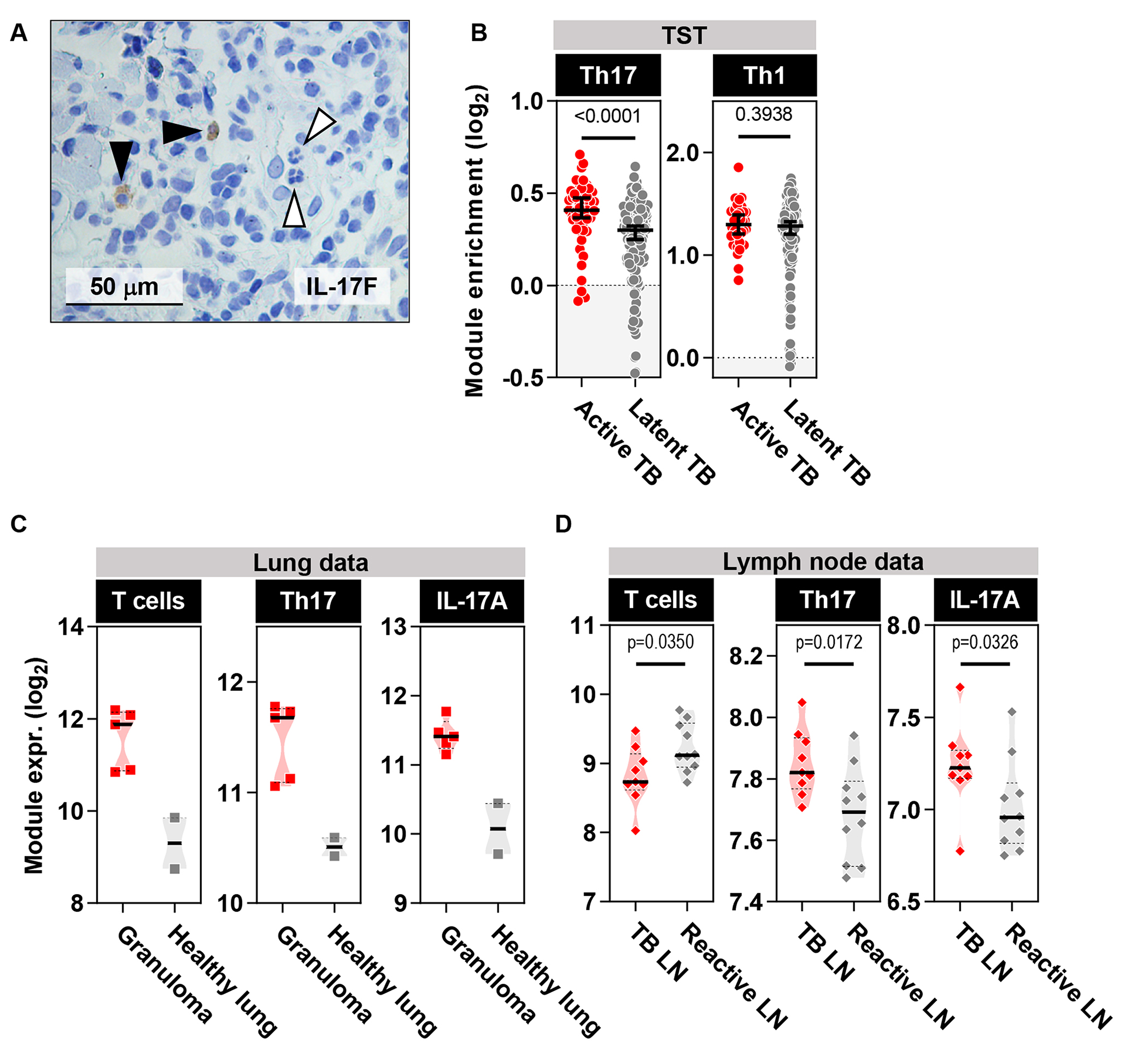
Active TB is characterized by elevated Th17 cells at the site of TST. **(A)** IL-17F immunohistochemistry (brown) in TST of patients with active TB. Black arrowheads point to mononuclear cells that express IL-17F and white arrowheads point to polymorphonuclear cells that do not express IL-17F. **(B)** Th17 and Th1 cell module enrichment in TST samples relative to saline injection in patients with active or latent TB. Analyses were performed on samples from 48 and 191 participants with active and latent TB, respectively. Horizontal lines and error bars on scatter dot plots represent medians with 95% confidence interval. **(C and D)** Expression of T cells, Th17 cells, and IL-17A bioactivity modules from the site of human TB granulomata (n=5) relative to healthy lung tissue (n=2) (dataset GSE20050) (C) and in human Mtb-infected lymph nodes (LN) (n=9) relative to reactive lymph nodes that do not display evidence of granulomatous inflammation or cancer (n=10) (D) (dataset E-MTAB-2547). Violin plots represent frequency distribution of all samples, with bold lines representing median values. All p values were calculated by Mann-Whitney tests.

IL-17A/F are predominantly produced by conventional Th17 cells, gamma delta (γδ) T cells and neutrophils (11). Immunohistochemistry revealed that in the TST of people with active TB, IL-17F, originated predominantly from mononuclear cells, rather than from polymorphonuclear cells (Fig. 4A). Therefore, we tested the hypothesis that Th17 cells were enriched in the TST of people with active TB compared to latent TB. We derived transcriptional modules specific for differentially polarized CD4^+^ Th subsets from a published dataset (23) (Data File S2) and demonstrated their specificity in an independent dataset (24) (fig. S4A and B). We further validated the Th17 module within skin biopsies of patients with psoriasis vulgaris, representing an alternative Th17-mediated inflammatory condition (25), showing that the transcriptional response to IL-17A positively correlated with the expression of the Th17 module (fig. S4C). Patients with active and latent TB revealed comparable expression of a Th1-associated transcriptional module, whereas expression of the Th17 module was increased in patients with active TB (Fig. 4B).

### IL-17A functional activity and Th17 cells are present at the site of TB disease

Having established that upregulation of IL-17A/F and its downstream functional activity was exaggerated in the tissue recall response to Mtb among people with active TB compared to those with latent TB, we sought to extend previous reports that IL-17A/F activity was also evident at the natural site of TB disease as previously described (8, 26). The transcriptome of human TB granuloma (27) showed an enrichment of T cells, Th17 cells and IL-17A induced genes compared to normal lung tissue (Fig. 4C). In addition, we examined the transcriptome of human Mtb-infected lymph node samples compared to unspecified ‘reactive’ causes of lymphadenopathy devoid of granulomatous inflammation or malignancy (28). We observed enrichment for our pan-T cell transcriptional module in reactive lymph nodes compared to TB lymphadenitis; however, the Th17 module and the module of IL-17A-induced genes was enriched within Mtb-infected lymph nodes (Fig. 4D). Overall, our data establish that an increased Th17 transcriptional signature and elevated IL-17A activity, which we observed in the TST in active TB, are also evident at the site of TB disease.

### Impact of curative antibiotic treatment, disease burden and demographics on IL-17A activity in active TB

**Figure 5:**
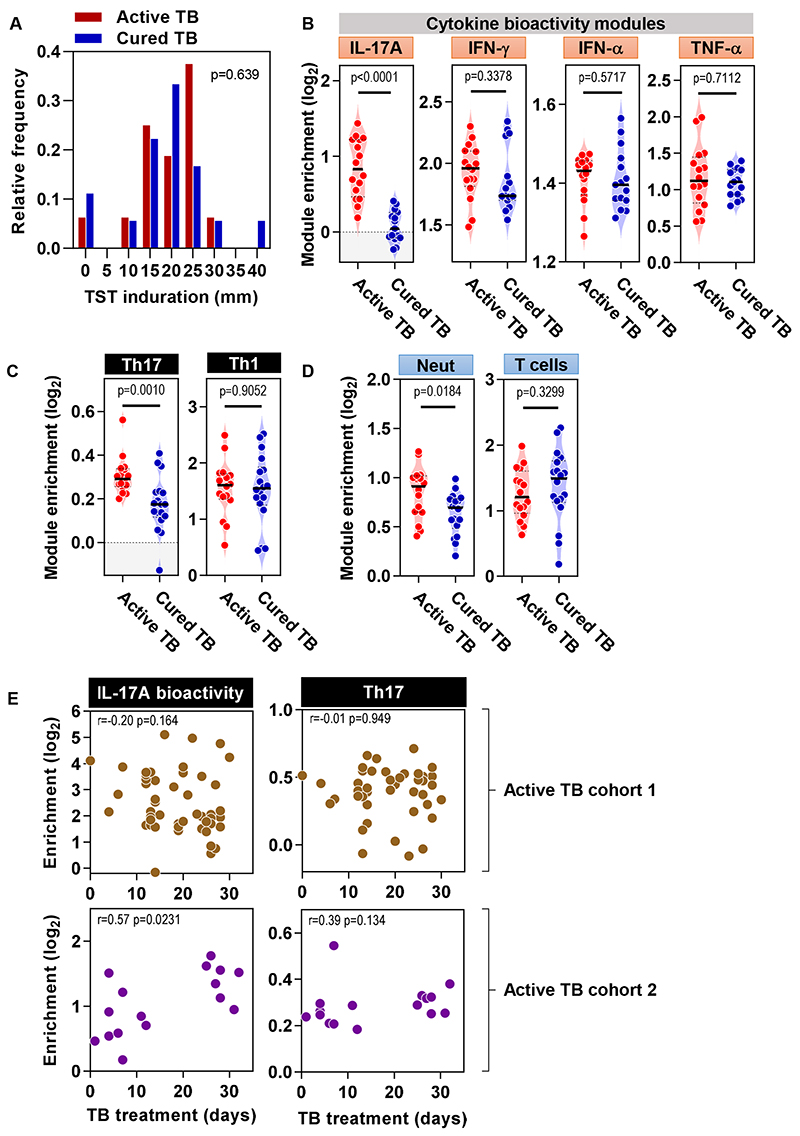
Complete antibiotic treatment for active TB attenuates IL-17A and Th17 responses at the site of TST. **(A)** Induration at the site of TST was recorded in millimeters by routine clinical assessments in patients with active and cured TB. **(B-D)** Enrichment in TST samples relative to saline injection was observed for keratinocyte response modules of cytokine bioactivity (B), Th17 and Th1 modules (C) and neutrophils and T cell modules (D). (A-D) Analyses performed on samples from 16 and 18 participants with active and cured TB, respectively. All p values were calculated by Mann-Whitney tests. Violin plots represent frequency distribution of all samples, with bold and dashed lines representing median and quartile values. All p values in (A-D) were calculated by Mann-Whitney tests. IL, interleukin; IFN, interferon; TNF, tumor necrosis factor. **(E)** The relationship in active TB cohorts between either IL-17A cytokine bioactivity or Th17 module enrichment and days on TB treatment prior to TST assessment was evaluated. 48 and 16 active TB patients were assessed in cohorts 1 and 2, respectively. r and p values determined by Spearman rank correlation.

We hypothesized that exaggerated IL-17A/F immune responses at the site of host-pathogen interactions in active TB may represent a maladaptive immune response associated with chronic immune stimulation arising from the failure to clear Mtb. To address this question experimentally, we tested the hypothesis that curative treatment for active TB would reverse the exaggerated IL-17A/F transcriptional responses in the TST challenge. We compared TST responses in a second cohort of patients with active TB in the first month of treatment (‘active TB’ cohort 2) to a second cohort of patients within two years of curative TB treatment (‘cured TB’) (Table 1). TST response induration was comparable between these cohorts (Fig. 5A). However, consistent with our hypothesis, the TST of cured TB patients showed reduced expression of *IL17A* and *IL17F* genes (fig. S5A), IL-17F protein (fig. S5B and C) and IL-17A inducible genes, as a measure of functional cytokine bioactivity (Fig. 5B). In addition, Th17- and neutrophil-associated transcriptional modules also showed lower expression in cured TB (Fig. 5B and C, fig. S3). Likewise, MMP1 gene expression and protein at the site of inflammatory infiltrates in the TST were also reduced in cured compared to active TB (fig. S5D to G). In contrast, there was no difference in expression of *IFNG* or *TNF* genes, in functional activity of IFN-γ, type I IFN, or TNF-α, or in expression of pan-T cell or Th1-associated transcriptional modules (Fig. 5B to D and fig. S5A).

Rapid changes in the peripheral blood transcriptome associated with active TB have been reported (29). Therefore, we tested the hypothesis that IL-17A inducible responses in the TST would also be diminished rapidly. TST sampling in active TB patients was performed at different time points within the first month of treatment (median 19 days, IQR 13-26 for cohort 1 and median 11 days, IQR 5-28 days for cohort 2). We did not observe a decrease in IL-17A bioactivity or Th17 responses over this time period of TB treatment (Fig. 5E). These findings suggest that the mechanisms that drive exaggerated IL-17A bioactivity in active TB persist for at least one month after the initiation of curative treatment. Participants with cured TB were assessed at different time points following completion of TB treatment, but we found no relationship between this time interval and functional IL-17A bioactivity or Th17 accumulation in the TST (fig. S6), indicating resolution of the processes that drive exaggerated IL-17A responses by completion of treatment.

We also considered whether the induction of IL-17A responses was associated with the burden of TB disease. For active TB patients in cohort 1, we had access to radiology and microbiology data, but found no association with radiological severity scores or microbiological load at the time of diagnosis (fig. S7). Similarly, there was no association between Th17 module or MMP-1 gene expression and these measures of TB burden (fig. S7). In addition, whereas all patients in active TB cohort 1 had pulmonary TB, cohort 2 included patients with extrapulmonary disease, but we found no difference in IL-17A or Th17 responses between people with pulmonary and extrapulmonary TB (fig. S8). Furthermore, in the larger cohort 1, sensitivity analyses revealed elevated IL-17A activity and Th17 responses in active TB to be independent of sex (fig. S9A) and ethnicity (fig. S9B). Active TB cohort 1 and the comparator latent TB cohort were all recruited from the United Kingdom (UK), but active TB cohort 2 consisted of patients from the UK and South Africa. There was no difference in IL-17A and Th17 responses among active TB patients recruited from the geographically distinct sites (fig. S10A). In a further sensitivity analysis among active TB cohort 2 restricted only to patients recruited from the UK, there remained differences in these responses among patients with active and cured TB (fig. S10B).

### Drivers of elevated IL-17A activity in active TB

**Figure 6:**
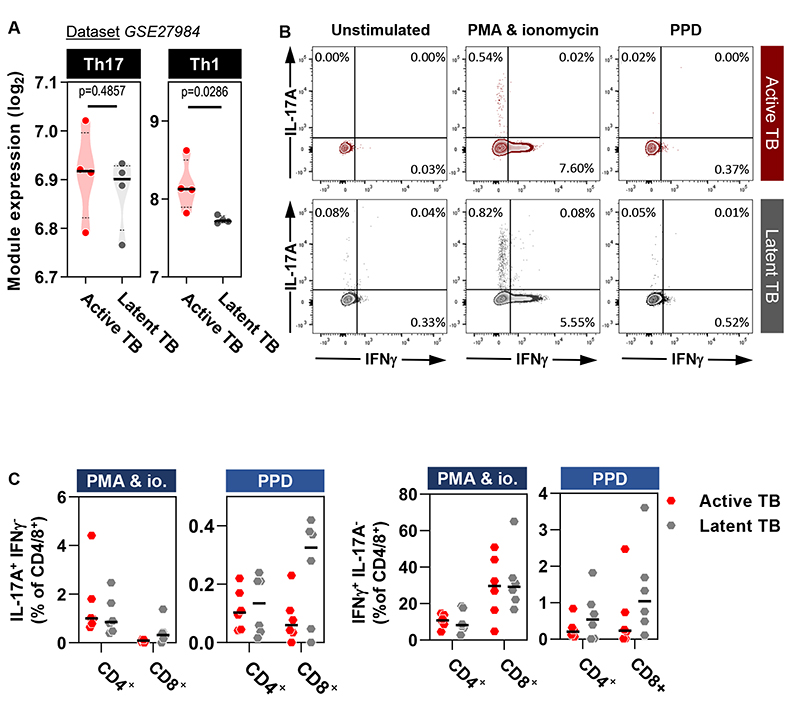
Active TB is not characterized by enrichment of Th17 cells in blood. **(A)** Expression of Th17 and Th1 modules in PPD-stimulated PBMC from patients with active or latent TB (n=4 in both groups) (data originated from dataset GSE27984). Violin plots represent frequency distribution of all samples, with bold and dashed lines representing median and quartile values. **(B)** Contour plots of CD3^+^CD4^+^ T cell intracellular staining for IFN-γ and IL-17A following PMA & ionomycin or PPD stimulation. Quadrant percentages represent proportion of total CD3^+^CD4^+^ T cell population. Top row (red) and bottom row (grey) are representative individuals with active and latent TB, respectively. **(C)** Summary data from experiments performed in (B), demonstrating proportion of IL-17A or IFN-γ expressing CD3^+^CD4^+^ or CD3^+^CD8^+^ T cells following stimulation with either PMA & ionomycin (io.) or PPD. Each dot represents an independent experiment. Horizontal black lines reflect median values for each group. Analyses performed on samples from 6 individuals each with active or latent TB from cohort 1. There were no statistical differences between any group by Mann-Whitney test.

To investigate the mechanism for increased Th17 responses in the TST of patients with active TB, we tested the hypothesis that disease was associated with increased frequency of circulating Th17 cells. We assessed the expression of the Th1 and Th17 subset modules in the transcriptome of peripheral blood mononuclear cells (PBMC) from individuals with active TB disease or individuals with latent TB, stimulated overnight with Mtb purified protein derivative (PPD) (30). In contrast to the evident differences at the site of the TST, expression of the Th17 associated transcriptional module in PBMC was not different between groups (Fig. 6A). We extended this analysis by performing intracellular cytokine staining in PBMC from subsets of active and latent TB individuals from cohort 1 following stimulation with PPD or nonspecific T cell activation with phorbol 12-myristate 13-acetate (PMA) and ionomycin. Neither stimulus revealed a propensity for CD4^+^ or CD8^+^ T cells in active TB to secrete greater amounts of IL-17A or IFN-γ (Fig. 6B and C). We interpret these results to mean that increased functional IL-17A bioactivity in the TST of patients with active TB was not due to increased recruitment of circulating T cells already committed to Th17 differentiation.

**Figure 7:**
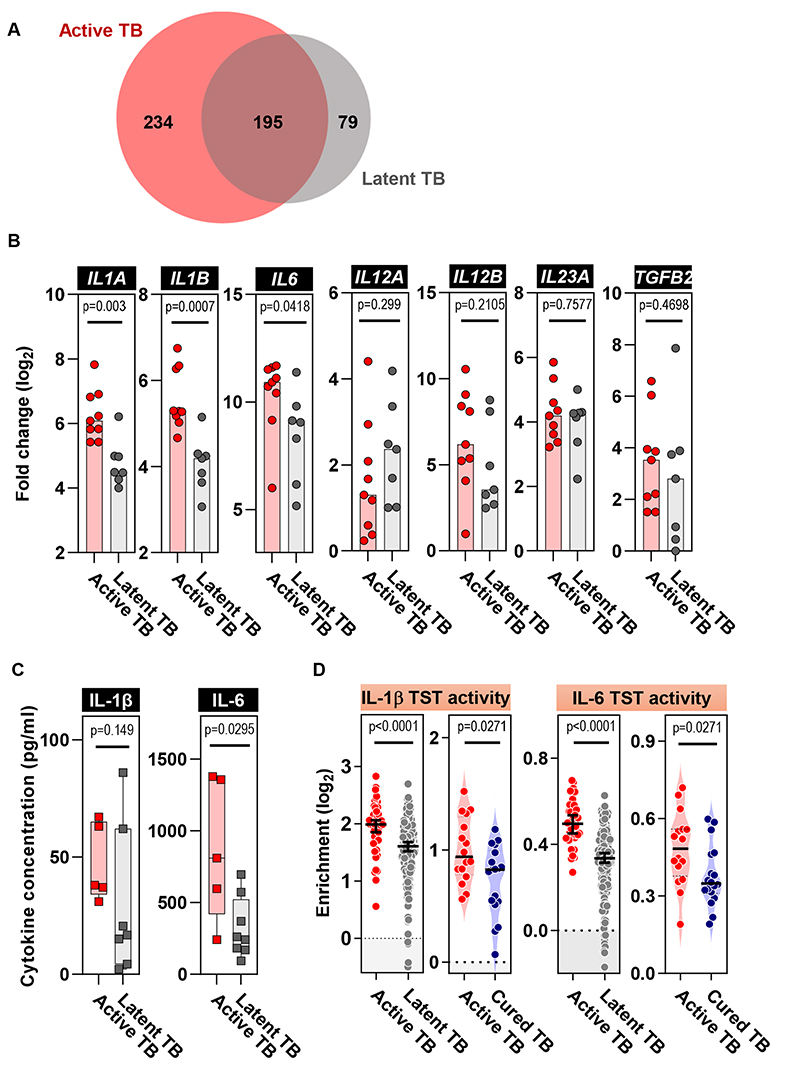
Elevated IL-1β and IL-6 induction by monocytes and cytokine activity in the TST in active TB. **(A)** Venn diagram depicting genes significantly upregulated in PPD-stimulated monocytes relative to unstimulated monocytes from patients with active or latent TB (Mann-Whitney test with FDR < 0.05). **(B)** Fold change induction of selected cytokine transcripts in monocytes in patients with active (n=9) or latent TB (n=7) following PPD stimulation. **(C)** Quantitation of IL-1β and IL-6 secretion by ELISA from monocytes isolated from patients with active or latent TB following PPD stimulation. All samples plotted, with boxes representing interquartile range and whiskers representing the full range of data. **(D)** Enrichment in TST relative to saline injection of IL-1β and IL-6 cytokine response modules. Analysis performed on samples from 48 and 191 participants with active or latent TB, respectively. Horizontal lines and error bars on scatter dot plots represent medians with 95% confidence interval. All p values were calculated by Mann-Whitney tests. *IL1A*, Interleukin 1A; *IL1B*, Interleukin 1B; *IL6*, Interleukin 6; *IL12A*, Interleukin 12 Subunit Alpha; *IL12B*, Interleukin 12 Subunit Beta; *IL23A*, Interleukin 23 Subunit Alpha; *TGFB2*, Transforming Growth Factor Beta 2.

Next, we explored the hypothesis that active TB may result in an increase in monocytes which could drive Th17 differentiation within the tissue response modelled by the TST. Several studies have demonstrated that systemic inflammatory perturbation, including Bacillus Calmette-Guérin (BCG) vaccination, can condition monocytes to differentially respond to secondary stimuli (31, 32). To test this hypothesis, we compared primary transcriptional responses of CD14^+^ monocytes to 4 hours PPD stimulation from individuals with active and latent TB. The baseline transcriptome of these cells did not identify any differences between the two groups, but following PPD stimulation, the transcriptional responses diverged (fig. S11A), inducing the upregulation of almost twice as many genes in active TB compared to latent TB (Fig. 7A and Data File S1). These included *IL1A*, *IL1B* and *IL6* genes, encoding cytokines that promote Th17 differentiation (33) (Fig. 7B). These findings were replicated by qRT-PCR (fig. S11B) and complemented by increased secretion of IL-6 protein in monocytes from patients with active TB (Fig. 7C). Consistent, with these findings *ex vivo*, we observed increased functional IL-1β and IL-6 cytokine activity in the TST of patients with active TB compared to either latent or cured TB (Fig. 7D), using transcriptional modules composed of IL-1β (fig. S12 and Data File S2) (34, 35) and IL-6 (36) inducible genes. Taken together, these data are consistent with a model in which exaggerated Th17 responses at the site of host-pathogen interactions in active TB are driven by recruitment of IL-1β or IL-6 producing monocytes (fig. S13).

## Discussion

We combine a standardized human antigen challenge model in which we are able to measure the functional activity of specific immune response pathways, with two independent cohorts of active TB and two separate comparator populations of latent and cured infection, to provide compelling evidence that in vivo IL-17A/F responses are exaggerated in active disease. Our findings suggest an important role for these responses in the immunopathogenesis of TB, which may be amenable to therapeutic targeting.

IL-17A/F contribute to host defence against bacterial and fungal pathogens, as demonstrated by mice deficient for IL-17A/F or IL-17 receptors, and by humans with inborn errors of IL-17 immunity (16). They are also strongly implicated in the immunopathology of chronic inflammatory diseases, such as psoriasis (16, 37). Animal models suggest a protective role for IL-17A/F following BCG vaccination (38) and in the early stages of Mtb infection (39–42) by promoting T cell chemotaxis to lymphoid follicles in Mtb-infected lungs (39) and by preventing formation of necrotic granuloma (43). However, in mice rendered susceptible to TB disease as a result of IFN-γ deficiency, and in mice receiving repeated BCG vaccinations to model persistent stimulation, IL-17A/F responses drive neutrophil-mediated pathology (12, 17–19). Furthermore, IL-17A/F can promote S100A8/9 and MMP-1 secretion by stromal cells (44, 45) and is strongly associated with neutrophil infiltration in chronically inflamed psoriatic lesions (46). We infer that IL-17A/F responses may play a dichotomous role in TB, contributing to protection in early infection, but driving pathology when the infection is not controlled and bacterial replication promotes chronic immune stimulation. Additional support for this model is evident in IL-27R deficient mice, which exhibit exaggerated Th17 responses because they lack IL-27 inhibition of RORγT (47). These mice show enhanced clearance of Mtb, but also increased immunopathology dependent on IL-17A. Interestingly, a human candidate gene study identified host genetic variation associated with increased secretion of IL-17A to be correlated with both protection from incident TB but also more severe TB disease (48). Taken together, we hypothesize that exaggerated IL-17A/F recall responses in active TB are the consequence of chronic multibacillary infection that mediates increased pathology in established TB disease.

Evolutionary conservation of Mtb-targeting T cell epitopes strongly suggests that T cell responses promote immunopathology (49), and that their negative regulation is critical to preventing disease manifestation (50). Our data support the hypothesis that exaggerated IL-17A/F responses arise from Th17 cells, but they do not unequivocally exclude other cellular sources. Using both transcriptional profiling and flow cytometry of circulating cells in blood, we did not observe evidence for increased circulating Mtb-reactive Th17 cells in active TB, consistent with most other studies (51–53). We note one previous report of increased concentrations of NKT cell derived IL-17 protein in active TB that reduced following treatment (54). We did observe Th17 cell enrichment and IL-17A bioactivity at the site of TB disease, also consistent with other reports (8, 26, 41, 42, 52). We note that some studies have not been able to detect increased IL-17 protein or Th17 cells from the inflammatory exudate associated with pericardial and pleural TB (51, 55). We speculate that these discrepancies may arise from inadequate sensitivity of the approach used to detect IL-17 cytokines and Th17 cells, or biological confounding related to the site of disease or the timing of sampling. Overall, however, our findings are supported by the majority of previous literature and suggest a model whereby differential Th17 responses observed within the TST are not governed by increased frequency of circulating Th17 cells, but by immune signalling networks within the tissue microenvironment after T cell recruitment.

We implicate circulating CD14^+^ monocytes as key players in promoting IL-17A responses at the site of host-pathogen interactions. We propose that infiltrating monocytes from patients with active TB produce elevated concentrations of cytokines, particularly IL-1β or IL-6, known to promote the differentiation of T cells to a Th17 phenotype (33). Trained immunity is increasingly reported to modulate immune responses by myeloid cells post-BCG vaccination (31, 32), but this was not seen in unstimulated myeloid progenitors following systemic Mtb infection in mice (56). We did not see differences in the baseline transcriptome of monocytes in active TB that might be expected as a result of epigenetic modifications associated with trained immunity. Nonetheless, modulation of transcriptional responses was observed following mycobacterial stimulation. This included increased IL-1β and IL-6 responses that have been implicated in both trained immunity and Th17 differentiation (31, 33, 57, 58). The mechanisms regulating this conditioned response in active TB will require further investigation to evaluate the potential roles of epigenetic and metabolic reprogramming (31, 57), and that of bacterial factors such as ESX-1 mediated secretion of ESAT-6 known to promote IL-6 and Th17 responses (59–61). However, we propose that the same molecular responses that are beneficial in the early stages of infection become dysregulated by the unresolved focus of infection in active TB disease, ultimately driving IL-17A/F-mediated immunopathology. IL-1 responses contribute to protection against Mtb challenge in mouse models (62), but exaggerated IL-1 and IL-6 responses promote immunopathology in other chronic inflammatory conditions, such as juvenile idiopathic arthritis and rheumatoid arthritis (36). Of note, increased TB disease severity has been linked to genetic polymorphisms associated with elevated IL-1β and IL-17A secretion (48, 63), as well as increased activity of the IL-6 axis in vivo (64).

The large active TB sample size in cohort 1 allowed us to explore and exclude contributions from sex and ethnicity. Despite the smaller sample size, we observed no differences in IL-17 activity between pulmonary and extrapulmonary TB in cohort 2, but these analyses will require validation in larger populations. We also observed no relationship between IL-17A responses and disease burden, as determined by plain chest radiography or sputum bacterial load. However, variation in both measures of severity is subject to confounding by duration of illness and may represent the volume of diseased tissue rather than the degree to which immune responses are skewed towards pathogenesis. Nevertheless, the maintenance of elevated IL-17A activity in the first month of treatment demonstrates that dynamic immune responses measured in our experimental model are independent of the immediate immunological changes that follow the onset of treatment, reflected in blood transcriptomic data (29). A future research priority will be to identify the point at which the effect of disease on IL-17A/F responses is reversed during recovery with antimicrobial treatment, in order to define a therapeutic window for host-directed immunomodulation in active TB disease. Sampling the TST response at time points beyond 48 hours may also provide insights into additional differences in homeostatic pathways between active and latent TB.

Our study has several limitations. The cross-sectional nature renders our observations susceptible to unmeasured confounding variables. We negated these risks through the inclusion of multiple cohorts to corroborate the generalizability of our findings. We have not established a causal association between exaggerated IL-17A responses and disease pathology or between elevated IL-1β or IL-6 monocyte responses and IL-17A bioactivity at the site of host-pathogen interaction. These associations are supported by multiple studies in the literature (12, 17–19, 23, 33, 65, 66), but in vivo experiments in non-human primates and interventional studies in humans are required to test the functional relationship between these cytokines in TB disease. Our bioinformatic analysis of transcriptional changes in tissues was validated by independently derived transcriptional signatures to enumerate immune cells and functional cytokine activity in vivo. Nonetheless, cellular- and protein-level data to further corroborate our findings will be sought in future work. In addition, we were not able to establish the relative contribution made by specific lymphocyte populations, such as γδ T cells or type 3 innate lymphoid cells (ILC3), to IL-17A/F expression as previously reported (40, 42, 54, 67, 68). Future studies will require single cell resolution, using single cell sequencing or multiplex RNA FISH assays, to confirm the source and spatial organisation of exaggerated IL-17A/F responses in this model, as well as to determine whether active TB shows enrichment for ‘pathogenic’ Th17 cells that express both IFN-γ and IL-17A/F (69).

Taken together, our data implicate IL-17A immune responses as drivers of tissue immunopathology in TB. We propose that therapeutic targeting of these responses as an adjunct to antimicrobial therapy in active TB offers strategies to ameliorate tissue damage and further reduce transmission of infection by promoting immune homeostasis. The availability of therapies that block IL-17A/F cytokine pathways, or upstream signals such as the IL-1α/β and IL-6 axes, (37, 70), offers invaluable opportunities to transition from proof of concept pre-clinical studies to first-in-human experiments. These studies are needed to establish the functional interaction between these cytokines and their causal role in the pathogenesis of human TB as a prelude to clinical trials for therapeutic benefit.

## Methods

### Study design

The aim of the study was to identify immunopathological responses in active TB. We hypothesized that in individuals with active TB, the tissue recall response to Mtb antigens using the standardized human TST challenge model (4–7), would be enriched for immune responses that drive tissue damage. The first objective was to identify cytokines that regulated the expression of genes over-expressed in the TST in active TB compared to non-diseased latent TB. Having identified IL-17A/F to be this cytokine, the second objective was to validate this finding experimentally by independently generating transcriptional modules that represented the cellular response to cytokine stimulation, and then demonstrating elevated IL-17A/F responses in the TST in active TB. The third objective was to define the potential source of IL-17A/F by generating modules for different CD4 T helper cell phenotypes and demonstrating enrichment for Th17 cells in active TB. Our fourth objective was to demonstrate that IL-17A/F responses were a feature of tissue responses at the site of active TB disease, making use of the transcriptome of Mtb infected lung granuloma and lymph nodes. Our fifth objective was to determine the role of curative antibiotic treatment on attenuating elevated IL-17A/F responses observed in active TB. Finally, our sixth objective was to identify putative mechanisms that drove elevated Th17 cell enrichment and IL-17A/F responses. We tested the hypothesis that circulating monocytes in active TB were conditioned to induce greater IL-1β and IL-6 responses following Mtb antigen stimulation, generating a local tissue environment that favoured Th17 differentiation at the site of host-pathogen interaction in TB disease.

Sample sizes are provided in Table 1 and are larger than those used to identify and reproduce differences in gene expression between study populations using the TST model in our previous studies (4, 5, 7). Recruitment to the study was performed strictly according to the inclusion criteria set out in table S1, and no randomisation was necessary. Details of experimental replicates are provided in accompanying figures. Gene expression analyses were not performed blinded, but all immunostaining and quantification was performed by investigators blinded to the study groups being compared.

Active TB cohort 1 was recruited from TB clinics in London, UK (figs 1-4 & 6). This population was compared to individuals with latent TB recruited from TB clinics in London, UK and Lima, Peru. Active TB cohort 2 that formed the basis of the analyses in figs 5-6 was the HIV seronegative cohort described in our previous publication (5), recruited from TB clinics in London, UK and Cape Town, South Africa. The comparator ‘Cured TB’ group was an independent cohort recruited from TB clinics in London, UK and Lima, Peru. Inclusion/exclusion criteria and characteristics of the study groups are described in table S1. On recruitment to the study, all participants provided a peripheral blood sample and underwent a tuberculin skin test or control saline injection, and the extent of inflammatory induration was measured at 48 hours and then subjected to two adjacent punch biopsies for transcriptional profiling or histology as previously described (5, 6). Radiological severity scores were derived according to established protocols (71).

The recruitment of patients with active, cured and latent TB was approved by UK National Research Ethics Committees (reference numbers: 14/LO/0505, 16/LO/0776 and 18/LO/0680), University of Cape Town Human Research Ethics Committee (reference number: 580/2012). and by Universidad Peruana Cayetano Heredia Institutional Ethics Committee (reference number: 62349) and was subject to written informed consent.

### TST sample processing for transcriptional profiling

Tuberculin skin tests (TSTs) were performed and biopsies from the site of TST were collected as previously described (5). Skin samples from all participants were stored in RNAlater at −70°C after collection. For processing, TST samples were equilibrated to room temperature for 30 minutes before being transferred to CK14 lysing kit tubes (Bertin Instruments) containing 350εI of Buffer RLT (Qiagen) supplemented with 1% 2-Mercaptoethanol (Sigma). Tubes were pulsed for 6 cycles on a Precellys Evolution homogenizer (Bertin Instruments), each cycle consisting of 23 seconds of homogenization at speeds of 6300 revolutions per minute (rpm). Samples were rested on ice for 2 minutes between cycles. After homogenization, cellular debris and lysing beads were precipitated by centrifugation and RNA isolated from the supernatant using RNeasy Mini Kit (Qiagen) as per manufacturer’s instructions.

### Genome-wide transcriptional profiling and transcriptomic data repositories

Assessment of the transcriptome of TST and monocytes from patients in cohort 1 (active and latent TB) was performed by RNA-Seq. cDNA libraries were generated using the KAPA Hyperprep kit (Roche), and sequencing was performed on the Illumina Nextseq using the Nextseq 500 High Output 75 cycle kit (Illumina) according to manufacturers’ instructions, giving 15-20 million 41bp paired end- reads per sample. Mapping and generation of read counts per transcript were done using Kallisto (72). The R/Bioconductor package tximport was used to import the mapped counts data and summarize the transcript-level data into gene level data (73).

The TST transcriptome from active TB cohort 2 was derived directly from the data repository E-MTAB-3254 (ArrayExpress), and transcriptional profiling of individuals with cured TB was performed by microarray, as previously described (5).

### Whole-genome analyses

All transcriptomic data were log_2_ transformed. RNAseq data were presented as TPM (transcripts per million) to remove feature-length and library-size effects. Microarray data were cleaned and normalized as previously described (4, 5). All gene expression data were annotated with Human Genome Organisation Nomenclature Committee (HGNC) gene symbols. Principal component analysis (PCA) was performed as previously described (5). Differential gene expression was conducted in MultiExperiment Viewer v4.9 (http://www.tm4.org/mev.html), using Mann-Whitney tests with a FDR<0.05. Pathway analysis was performed in InnateDB using the Reactome Pathway Database, and visualized as network diagrams in Gephi v0.8.2 beta (5). Upstream regulator analysis was performed using Ingenuity Pathway Analysis (Qiagen), focusing on cytokines with predicted activation z-score >3. The expression of transcriptional modules within genome-wide data was determined by calculating the geometric mean expression of all the module constituent genes found in the dataset being analyzed, using R scripts generated in our previous publication (21), which are available to download and use from the Github repository (https://github.com/MJMurray1/MDIScoring). Venn diagrams were constructed using the BioVenn tool (http://www.biovenn.nl/).

### Gene expression module derivation and analyses

We used the immune cell modules immune cell modules “M37.1” (neutrophils), “M19” (T cells), “M69” (B cells) and “M7.2” (NK cells) that we previously that we previously identified to be most sensitive and specific for their respective annotations (21). Where indicated, relative neutrophil proportions were also estimated from TST samples using ABsolute Immune Signal (ABIS) deconvolution (22).

Keratinocyte (KC) cytokine-response modules were derived from published transcriptomic data (GSE12109 & GSE36287) of primary human KC stimulated with a selection of cytokines (14, 15). Significant transcriptional responses (paired t-test with α of p<0.05 without multiple testing correction) of genes over-expressed >4-fold in the cognate cytokine condition relative to unstimulated KC were initially identified. Genes that were also upregulated > 2-fold by non-cognate cytokines compared to unstimulated KC were excluded. The KC IL-17 response module utilized a cut-off of 2-fold between IL-17 stimulated KC and unstimulated KC as too few genes were upregulated >4-fold compared to unstimulated KC. The KC TNF-α response module generated in this way has already been published (6), but the other cytokine modules have not been previously described. The constituent genes of the KC modules are shown in Data file S2. Their specificity was evaluated in both the data from which they were derived and in independent data (GSE36287) of in vitro cytokine-stimulated KC (fig S2) (15).

‘Th’ subset modules were derived from published transcriptomic data of CD4^+^ T cells polarized towards different T helper phenotypes (GSE54627) (23). To derive specific modules for each differentiation state, we used gene expression from cells stimulated with anti-CD3 and anti-CD28. We initially identified an integrated list of genes induced in either Th1 or Th17 cells compared to unpolarized naïve T cells after anti-CD3 and anti-CD28 stimulation using a paired t-test with α of p<0.05 without multiple testing correction. Modules for each T helper phenotype were derived from this integrated list, identifying genes over-expressed in the phenotype of interest compared to all other conditions in the dataset (Th1, Th2, Th17 & T cells stimulated with IFN-γ). Each module was derived from the unique genes expressed >1.5-fold in the cognate condition compared to all other stimulation conditions. The gene components of Th1 and Th17 modules are available in Data file S2. Their specificity was evaluated in the dataset from which they were derived, in an independent dataset of polarized CD4 T cells, and in skin biopsies of patients with psoriasis vulgaris (fig. S4) (23–25).

An IL-1β-response module was derived from dataset cytokine stimulated fibroblasts (34) using an unpaired t-test with α of p<0.05 without multiple testing correction, and identifying genes induced >2-fold in IL-1β stimulated fibroblasts compared to TNF-α-stimulated fibroblasts. The gene components of this IL-1β bioactivity modules are available in Data File S3. The specificity of the module was evaluated in the dataset from which it was derived, in an independent dataset of IL-1β stimulated peripheral blood mononuclear cells (PBMCs) (Accession no: GSE40838) and in skin from individuals treated with recombinant IL-1 receptor antagonist, anakinra (Accession no: GSE27864) (35). An IL-6 response module was also used in the analyses presented (Fig 7). The derivation and extensive validation of this module was described previously (36).

### PBMC stimulation

PBMCs were obtained by density gradient centrifugation of heparinized whole blood with Ficoll-Paque PLUS (GE Healthcare Biosciences) plated at a concentration of 1x10^6^ cells/ml in RPMI-1640 supplemented with 5% heat-inactivated human AB sera for 16 hours, followed by 4 hours of stimulation with 10 μg/mL purified protein derivative (PPD) (SSI) or 25 ng/ml PMA (Sigma) and 1 μg/ml ionomycin (Sigma), in conjunction with 10 μg/mL brefeldin A (Sigma).

### Flow cytometry

PBMCs were stained with Live/Dead Fixable Blue Dead Cell Stain at a 1:1000 dilution (Invitrogen) for 30 minutes, and subsequently incubated with Human Trustain FcX (BioLegend) before cell surface staining at 4°C for 30 minutes with the following antibodies (all at a 1:100 dilution, obtained from BioLegend): CD3 (HIT3a), CD4 (SK3), CD8 (SK1). Cells were then fixed and permeabilized in Fixation Buffer and Intracellular Staining Permeabilization Wash Buffer (BioLegend) at 4°C for 20 minutes according to the manufacturer’s instructions. Quantification of intracellular cytokine was performed using antibodies to IL-17A (BL168) and IFN-γ (4S.B3), and associated isotype controls (all using a 1:100 dilution and obtained from BioLegend). Staining was performed at 4°C for 30 minutes. Flow cytometry was performed with LSR Fortessa X20 (BD Biosciences), and data were analyzed with FlowJo (Tree Star).

### Monocyte isolation and stimulation

In selected patients, PBMCs were isolated by density gradient centrifugation and CD14^+^ monocytes were isolated by positive selection magnetic cell sorting using CD14 MicroBeads (Miltenyi Biotec) as per manufacturer’s instructions (31). Monocytes were then cultured in RPMI-1640 supplemented with 5% pooled human AB serum (Sigma) with and without stimulation with 100ng/ml PPD (SSI) for 4 hours. Total RNA from monocytes was collected using RLT buffer (Qiagen) and isolated using the RNeasy Micro kit (Qiagen)as per manufacturer’s instructions.

### Immunostaining

Immunostaining of IL-17F was performed on 10 μm sections as previously described (74). Briefly, monoclonal mouse-anti-IL-17F at a 1:50 dilution (MA5-16229, Thermo Fisher Scientific) and 4′,6-diamidino-2-phenylindole (DAPI) (sc-24941, Santa Cruz Biotechnology) for detecting nuclei were used. Irrelevant primary antibodies from Sigma Aldrich (irrelevant mouse antibody, 1:50, code I8765) was applied at the same concentration of the related specific primary antibodies for immunostaining of negative control slides. Light-microscopic analysis was performed at a magnification of 40x with a Leica DM4000B microscope equipped with DFC-320 Leica digital camera (Leica Microsystems). Zeiss confocal microscope (LSM800) was used to acquire confocal images. Quantification of IL-17F was performed on 10x confocal images of samples through the measurement of mean grey value (MGV) using ImageJ (National Institutes of Health).

Immunostaining of MMP-1 was performed as previously described (75). Scanned slide images were obtained with use of NanoZoomer Digital Pathology System (Hamamatsu Photonics). Quantification of MMP-1 staining was performed blindly by extracting MMP-1 associated 3, 3- diaminobenzidine (DAB) stain using standard deconvolution protocols in ImageJ (NIH). Cellular infiltrates were manually selected as depicted in Fig 2 and DAB stain quantified by staining intensity as proportion of the area selected using ImageJ. The selected region was moved without resizing to adjacent tissue that did not contain cellular infiltration to calculate background MMP-1 intensity. Three cellular infiltrates and background tissue regions were analyzed for each tissue samples. Six TST samples were quantified in each group.

### Quantification of microbial load and radiographic scoring

Quantification of bacterial burden was estimated from two routinely used clinical diagnostic approaches: sputum smear scoring and the time for positive detection of *Mycobacterium tuberculosis* (Mtb) in liquid culture, a measure that is inversely associated with input bacterial count. Radiological severity scores were derived by estimating the percentage of the lung fields affected by abnormality, with an additional penalty for the presence of cavitation as previously described (71).

### Statistical analysis

On scatter and violin plots, horizontal lines represent median values, and error bars reflect 95% confidence intervals of median. Throughout the manuscript, values from two independent groups were compared by two-tailed Mann-Whitney test and p value of <0.05 was interpreted as significant. Where indicated, a false discovery rate (FDR) adjusted p value was used to account for increased alpha from multiple testing. Correlations between variables were determined using 2 tailed Spearman’s rank correlation analyses. Associations between patient cohorts’ ethnic background and disease group were determined by Chi-squared test, whereas associations for variables with only two categories (sex and site of TB disease) were determined by Fisher’s exact test. For both of these contingency analyses, we interpreted a p value of <0.05 to indicate a significant association between the variables.

## Supplementary Material

Supplementary materials

## Figures and Tables

**Table 1:** Patient cohort metadata. Comparisons of age, gender and TST induration between active and cured TB participants performed by Mann-Whitney tests. Comparison in gender, ethnicity and site of disease performed by Chi-square tests. TST, tuberculin skin test; N/A, not applicable; IQR, interquartile range.

Cohort 1 (figs 1 to 4)		TST			Saline controls
		Active TB	Latent TB	Active versus Latent	
**Number**		48	191		34
**Received treatment for latent TB prior to TST**		-	7		-
**Age (median & range)**		30.5 (19-77)	35 (18-65)	p = 0.16	28 (18-75)
**Gender**	Male (%)	63	46	p = 0.028	42
	Female (%)	37	54		58
**Ethnicity**	White (%)	43	27	p = 0.015	41
	Black (%)	23	23		19
	South American (%)	2	6		25
	Asian (%)	28	21		25
	Mixed (%)	4	22		6
**Site of disease**	Pulmonary (%)	100	N/A	-	-
**TST sampling from start of treatment (median & IQR)**		19 (13-26)	-	-	-
**TST induration (median & range)**		20 (0-70)	18 (0-47)	p = 0.07	0 (all)
					

Cohort 2 (Fig 5)		TST			Saline controls
		Active TB	Cured TB	Active versus Cured	
**Number**		16	18		10
**Age (median & range)**		40 (25-71)	43 (20-60)	p = 0.11	39 (18-54)
**Gender**	Male (%)	62	61	p = 0.88	70
	Female (%)	38	39		30
**Ethnicity**	White (%)	14	23	p < 0.001	20
	Black (%)	57	5		70
	South American (%)	0	50		0
	Asian (%)	19	11		10
	Mixed (%)	10	11		0
**Site of disease**	Pulmonary (%)	66	71	p = 0.45	90
	Extrapulmonary (%)	34	29		10
**TST sampling from start of treatment (median & IQR)**		11 (5-28)	-	-	-
**Days from end of treatment (median & range)**		-	76.5 (0-491)	-	-
**TST induration (median & range)**		20 (0-28)	19 (0-38)	p = 0.64	0 (all)

## Data Availability

All data associated with this study are in the paper or supplementary materials. All transcriptional datasets used in this study are described in Data File S3. Accession numbers refer to datasets in the ArrayExpress repository (https://www.ebi.ac.uk/arrayexpress/) or Gene Expression Omninbus (https://www.ncbi.nlm.nih.gov/geo/).

## One Sentence Summary

In vivo IL-17A activity is elevated in active tuberculosis and may be a therapeutic target to attenuate disease-associated immunopathology.
