# Supplementary material for: Exaggerated IL-17A activity in human in vivo recall responses discriminates active tuberculosis from latent infection and cured disease
Source: Sci Transl Med. Author manuscript; Available in PMC 2021 May 20. (PMC7610803; doi:10.1126/scitranslmed.abg7673)
Supplement: Supplementary materials [file EMS123945-supplement-Supplementary_materials.docx]

# Supplementary Figures

##
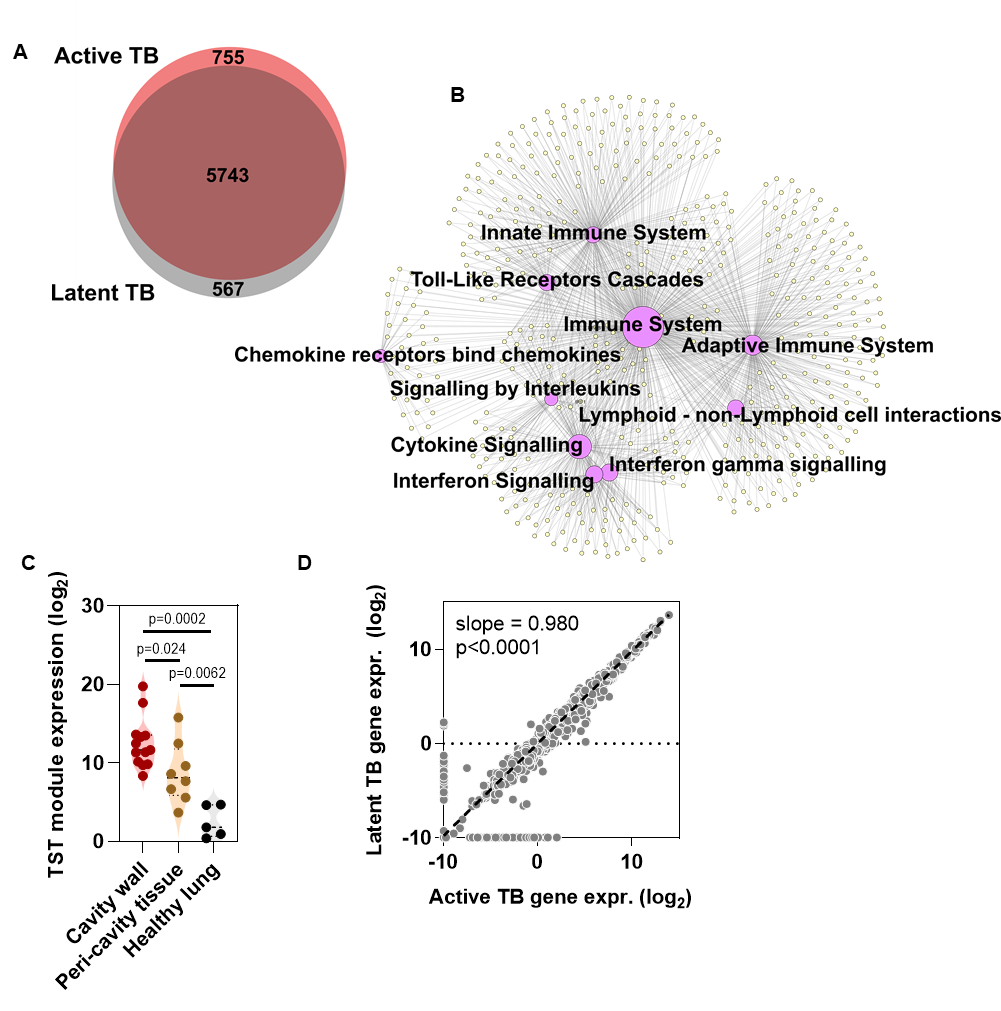


**Fig. S1. Overlapping and distinct TST transcriptional responses in active and latent TB.** **(A)** Venn diagram depicting number of genes significantly upregulated in the TST of patients with active or latent TB relative to control saline injection (Mann-Whitney test with false discovery rate <0.05). **(B)** TST transcriptome common to both active and latent TB summarized as a network diagram. Purple nodes represent Reactome database functional pathways, yellow nodes represent genes and edges reflect relationship between pathways and genes. Pathway node diameters are proportional to the respective pathway –log10 p value enrichment statistic. **(C)** TST responses shared between active and latent TB individuals were used to generate a module, and its expression was determined within the transcriptome of human Mtb-infected lung cavity walls, macroscopically-normal lung tissue adjacent to cavities and non-Mtb infected healthy lung tissue. All p values were calculated using Mann-Whitney tests. **(D)** Pairwise dot-plot of 5743 gene integrated TST signature in patients with either active or latent TB. Dotted line reflects line of perfect covariance. The indicated p value was derived from linear regression modeling between the two variables.

**
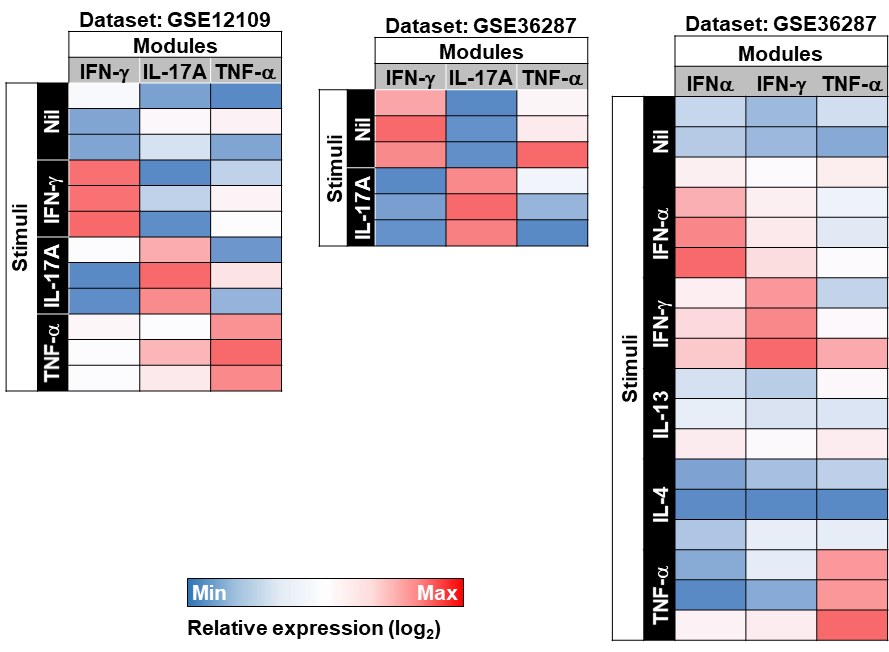
**

**Fig. S2. Assessment of sensitivity and specificity of cytokine-stimulated keratinocyte transcriptional response modules.** The expression of transcriptional modules derived from in vitro cytokine stimulation of keratinocytes (KC) determined in multiple datasets of in vitro stimulated KC. Heat maps reflect relative expression of each module (columns) in the experimental conditions (rows).

##


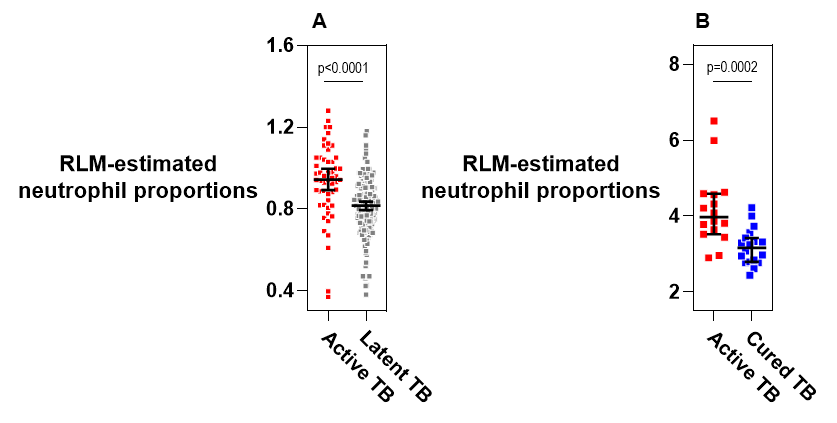


**Fig. S3. Deconvolution of neutrophil proportions in the TST in active, latent, and cured TB.** Neutrophil proportions were estimated using robust linear modelling (RLM) and cross-normalized absolute immune signatures (ABIS). The signature matrices ABIS-Seq and ABIS-microarray were used for **(A)** RNA-seq and **(B)** microarray data respectively. Analyses performed on samples from 48 and 191 participants with active or latent TB in **(A)** and on samples from 16 and 18 participants with active and cured TB in **(B)**. Horizontal lines and error bars on scatter dot plots represent medians with 95% confidence interval. All p values were calculated by Mann-Whitney tests.


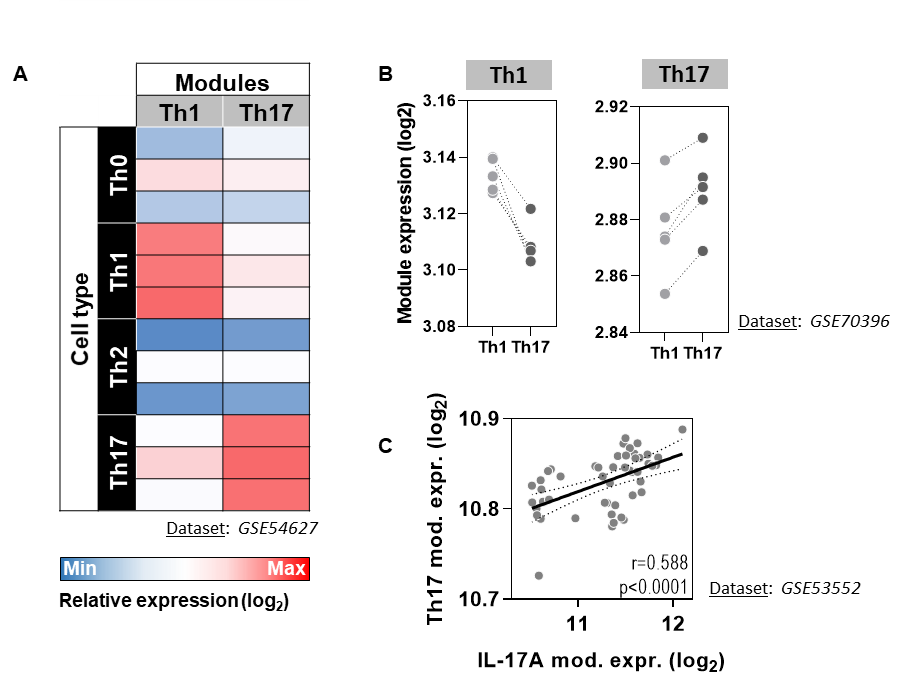


**Fig. S4. Assessment of sensitivity and specificity of polarized CD4+ T helper cell transcriptional modules.** **(A)** The expression of transcriptional modules derived from in vitro polarization of CD4+ T cells in datasets containing T helper polarized phenotypes. Heat maps reflect relative expression of each module (columns) in the experimental conditions (rows). **(B)** Expression of Th1 and Th17 modules in CD4+ Th1 and Th17 cells. Each pair of dots represents Th1 and Th17 cells from the same donor. **(C)** Relationship between the expression of IL-17A keratinocyte response module and Th17 module in the skin of patients with psoriasis vulgaris. r and p values were determined by Spearman rank correlation.

**
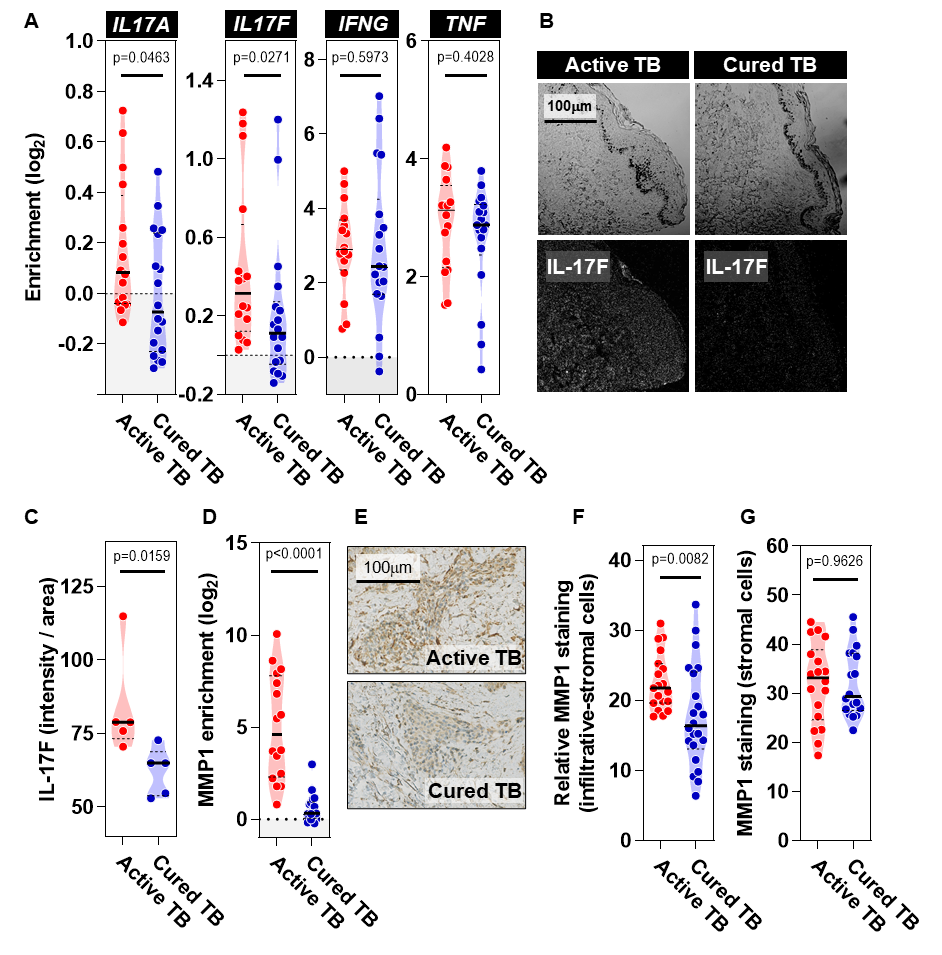
**

**Fig. S5. IL-17A/F and MMP-1 are enriched in the TST of individuals with active TB. (A)** Enrichment of selected gene transcripts within the TSTs of patients with active or latent TB relative to saline injection. **(B)** Expression of IL-17F by immunofluorescence in TSTs of patients with active or cured TB. The top panels show phase contrast images, and the bottom panels show IL-17F staining (white). **(C)** Quantification of IL-17F staining throughout TST sections from patients with active (n=5) or cured TB (n=5), determined by pixel intensity as a proportion of area sampled. Each dot represents IL-17F expression in the cross-section of an entire TST biopsy from one patient. **(D)** MMP1 gene mRNA enrichment in TST of patients with active (n=16) and cured TB (n=18) relative to saline injection. **(E)** Representative MMP-1 immunohistochemistry staining, quantified by DAB stain (brown), in inflammatory infiltrates from TST samples derived from patients with active or cured TB. Nuclei were counterstained using hematoxylin (blue). **(F)** Differential MMP-1 staining intensity for 18 cellular infiltrates from 6 patients in each group (3 infiltrates quantified per TST section) relative to adjacent zones of skin with no cellular infiltration. **(G)** MMP-1 staining intensity in TST zones outside inflammatory infiltrates. Violin plots represent frequency distribution of all samples, with bold and dashed lines representing median and quartile values. All p values were calculated by Mann-Whitney tests.

**
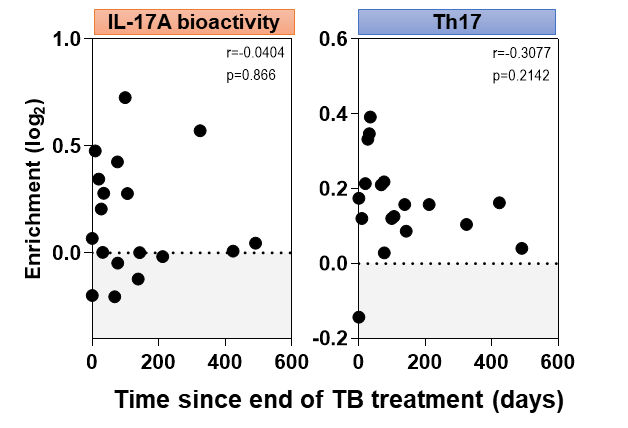
Fig. S6. IL-17A bioactivity and Th17 enrichment in TST from individuals with cured TB is not confounded by time since end of TB treatment.** Relationship between the time elapsed since participants with cured TB had finished TB treatment (days) and enrichment of either IL-17A keratinocyte transcriptional response or Th17 modules in TST relative to saline injection. r and p values were determined by Spearman rank correlation.

**
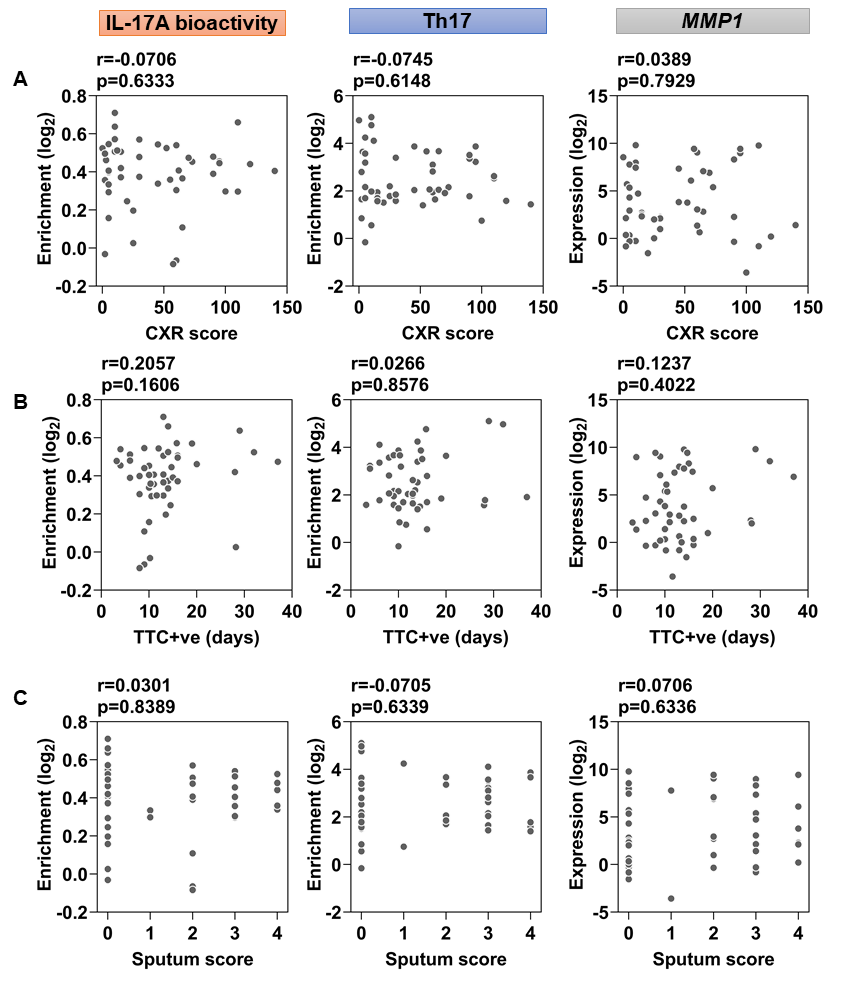
**

**Fig. S7. IL-17A bioactivity and Th17 infiltration in TST is not associated with radiological severity or microbiological load at the time TB disease diagnosis.** Enrichment of IL-17A keratinocyte transcriptional response and Th17 modules, or MMP1 gene expression, in TST of individuals with active TB relative to saline injection stratified by **(A)** chest X-rays (CXR) scores of radiological severity, **(B)** time to culture positivity (TTC+ve) for Mtb in liquid culture or **(C)** sputum smear scoring at the time of TB diagnosis. r and p values were determined by Spearman rank correlation.


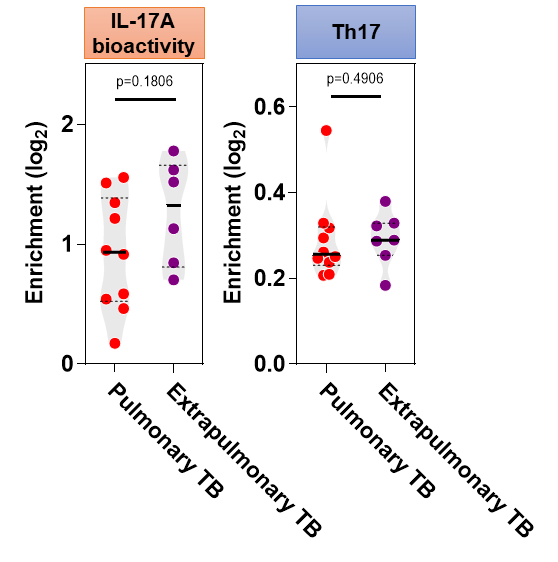


**Fig. S8. IL-17A bioactivity and Th17 cell enrichment in TST is not confounded by extrapulmonary TB disease.** Enrichment of IL-17A keratinocyte transcriptional response and Th17 cell modules in TSTs of patients with active TB with pulmonary and extrapulmonary TB relative to saline injection. Violin plots represent frequency distribution of all samples, with bold and dashed lines representing median and quartile values. All p values were calculated by Mann-Whitney tests.


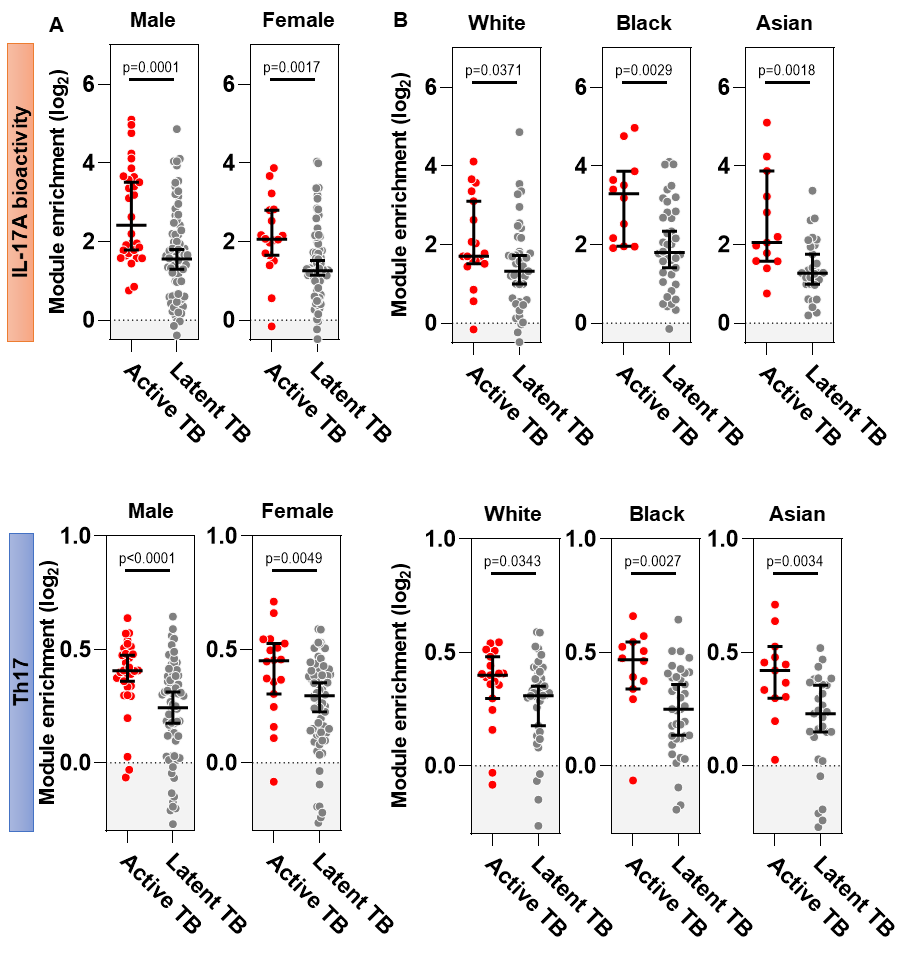


**Fig. S9. Differences in IL-17A bioactivity and Th17 cell enrichment by gender and ethnicity.** Enrichment in TSTs of active TB patients of IL-17A keratinocyte transcriptional response and Th17 cell modules by **(A)** sex or **(B)** ethnicity. Horizontal lines and error bars on scatter dot plots represent medians with 95% confidence interval. All p values were calculated by Mann-Whitney tests.


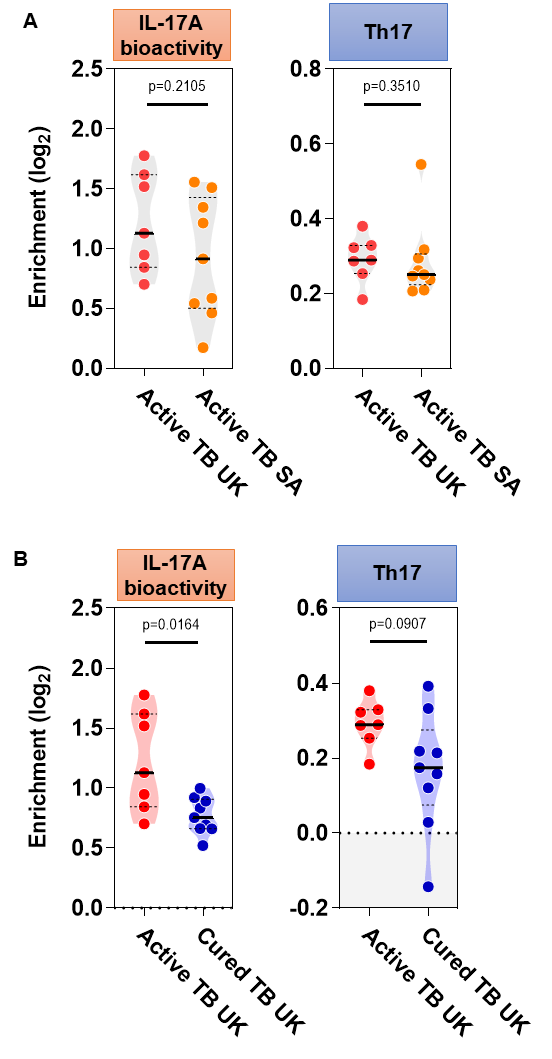


**Fig. S10. IL-17A bioactivity in active TB is not confounded by country of study recruitment.** Enrichment relative to saline injection of IL-17A keratinocyte transcriptional response and Th17 modules in TSTs from **(A)** individuals with active TB recruited in the UK and South Africa (SA), or **(B)** individuals with active and cured TB recruited solely from the UK. Violin plots represent frequency distribution of all samples, with bold and dashed lines representing median and quartile values. All p values were calculated by Mann-Whitney tests.


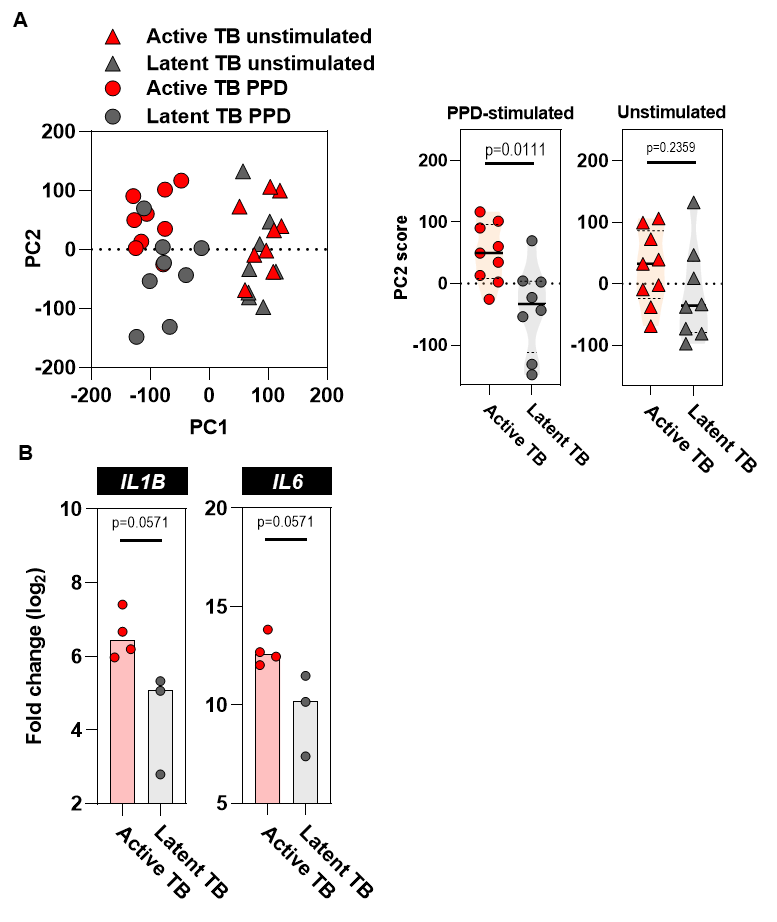


**Fig. S11. Monocyte transcriptional responses in active and latent TB.** **(A)** Principal component analysis (PCA) of monocyte transcriptome from patients with active or latent TB, at baseline or following PPD stimulation. Right hand panels reflect PC2 scores for PPD-stimulated or unstimulated monocytes in the patient groups. Violin plots represent frequency distribution of all samples, with bold and dashed lines representing median and quartile values. **(B)** Fold change induction following PPD stimulation of IL1B and IL6 genes in monocytes from patients with active or latent TB, as measured by qRT-PCR. All p values were calculated by Mann-Whitney tests.


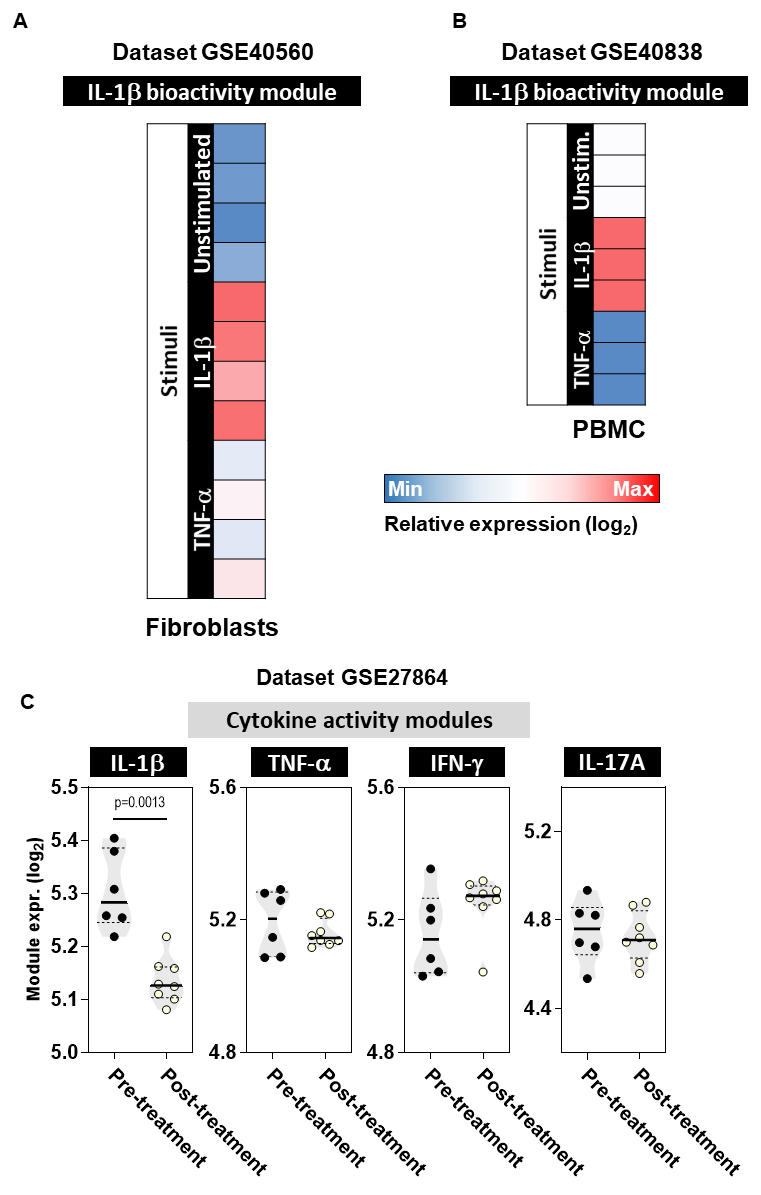


**Fig. S12. Assessment of sensitivity and specificity of IL-1β transcriptional response module.** The expression of IL-1β transcriptional response modules in **(A)** fibroblasts stimulated in vitro for 6 hours with IL-1β (10 ng/ml) or TNF-α (20 ng/ml), **(B)** PBMC from healthy volunteers stimulated in vitro for 6 hours with IL-1β (10 ng/ml) or TNF-α (20 ng/ml), and **(C)** skin biopsies from patients with neonatal-onset multisystem inflammatory disease (NOMID) before and after treatment with the IL-1 receptor antagonist, anakinra. Violin plots represent frequency distribution of all samples, with bold and dashed lines representing median and quartile values. All p values were calculated by Mann-Whitney tests.


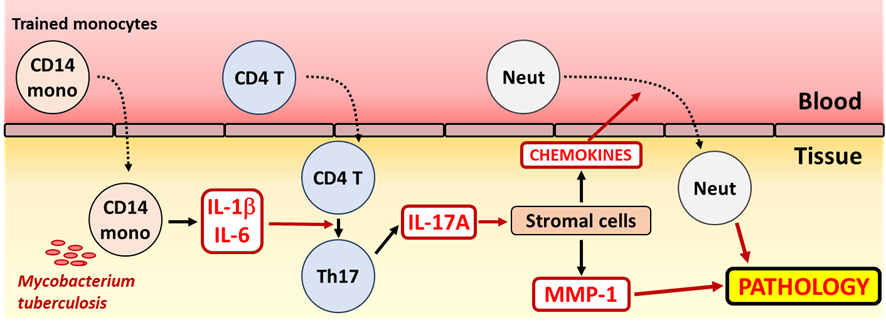


**Fig. S13. Proposed model for IL-17A-driven immunopathology in active TB.** In active TB, conditioned monocytes in circulation are recruited to Mtb-infected tissues and respond with elevated IL-1β and IL-6 production that promotes local differentiation of T cells to a Th17 phenotype. The ensuing exaggerated IL-17A bioactivity promotes MMP-1 production and neutrophil chemotaxis that contribute to tissue pathology.

**Table S1. Study groups' inclusion and exclusion criteria.**

| **Active TB patients** | |
| --- | --- |
| **Inclusion criteria** | **Exclusion criteria** |
| Microbiologically confirmed pulmonary TB | HIV seropositive |
| <4 weeks since starting TB treatment | Neoplastic disease |
| >16 years age | Hepatitis B/C co-infection |
|  | Immunomodulatory therapy (corticosteroids or interferon) |
|  | Immunization within preceding 2 weeks |
|  | Existing paradoxical reaction to anti-tuberculosis treatment |
|  | Previous keloid formation |

| **Latent TB patients** | |
| --- | --- |
| **Inclusion criteria** | **Exclusion criteria** |
| Positive peripheral blood IFN-γ release assay (IGRA) | Clinical or radiological evidence of TB disease |
|  | HIV seropositive |
|  | Neoplastic disease |
|  | Hepatitis B/C co-infection |
|  | Immunomodulatory therapy (corticosteroids or interferon) |
|  | Immunization within preceding 2 weeks |
|  | Existing paradoxical reaction to anti-tuberculosis treatment |
|  | Previous keloid formation |

| **Cured TB patients** | |
| --- | --- |
| **Inclusion criteria** | **Exclusion criteria** |
| Microbiological diagnosis of active TB | Clinical or radiological evidence of TB disease |
| M tuberculosis sensitive to at least 3 first line anti-TB drugs (rifampicin, isoniazid, pyrazinamide, ethambutol) | HIV seropositive |
| <24 months from completion of curative anti-TB treatment | Neoplastic disease |
| >18 years age | Hepatitis B/C co-infection |
|  | Immunomodulatory therapy (corticosteroids or interferon) |
|  | Immunization within preceding 2 weeks |
|  | Existing paradoxical reaction to anti-tuberculosis treatment |
|  | Previous keloid formation |
